# Modern Sociology

**Published:** 1896-10-17

**Authors:** 


### Modern Sociology.

### THE ROMAN INFLUENCE.

No more vivid, and, on the whole, more truthful description of the mission of Rome amongst the nations has ever been given than that embodied in the stirring lines of Macaulay—

> "Leave to the sons of Carthage
> The rudder and the oar;
> Leave to the Greek his marble Nymphs
> And scrolls of wordy lore.
> Thine, Roman, is the pilum;
> Roman, the sword is thine;
> The even trench—the bristling mound,
> The legion's ordered line."

The history of Rome is of all ancient secular history the most interesting and most important to the student of sociology, and for this reason—that all the ancient civilisations which have left any impress upon modern nations eventually became merged in that of Rome. The Roman Empire was founded upon the ruins of a hundred different polities—as a great modern historian has justly remarked. The city of Rome gradually extended its dominion over Italy, over Spain, and over Gaul; destroyed the Carthaginian Empire; became the protector of Greece, and the colonies that had been originally founded by Greeks; and finally ruled the East as far as the Euphrates and the mountains of the Caucasus. The finishing touch was put to this vast dominion when, in the year 211 A.D., the Roman citizenship was extended to all subjects of the Empire by the Emperor Antonius Caracalla. But before this stupendous task of world conquest and civilisation could be achieved, Rome for centuries was torn by internal conflicts between the patricians and plebeians, or, roughly speaking, between aristocracy and democracy. When a *modus vivendi* had been found between the conflicting claims of these two parties, the rule of foreign conquest began, and was continued uninterruptedly until the Romans might justly say that the whole civilised world was under the dominion of the Roman Senate and people. The political change had its counterpart in the Latin language. Two well-known words clearly express it. "Urbanitas" was the ideal of the old Roman gentleman; but presently the more comprehensive word, "Humanitis," took its place, and signified the broader spirit which underlaid the new cosmopolitanism. No such political phenomenon has appeared in modern times, nor indeed is likely to appear in the future. So far, since the break-up of the Roman Empire, all attempts at universal dominion have signally failed, whether attempted by a Charlemagne, by the Emperor Charles V., or, almost in our own times, by a Napoleon. There are now six so-called "Great Powers" in Europe alone.

But no one of these States holds the same position in the modern as Rome did in the ancient world. In the words of the poet—

> "Ambition's boldest dream and last
> Must melt before the clarion blast
> That sounds the dirge of Rome."

In connection with this aspect of the Roman power it is well to remember the attitude assumed by even the most enlightened Romans of the day towards Christianity. In a passage extracted from the chronicle of Sulpicius Severus, which a famous German critic considers might have been written by Tacitus, the great Roman historian, the author says, speaking of the events that happened immediately after the fall of Jerusalem, "Titus (who was afterwards Emperor), calling a council, considered the question whether he should destroy so vast an erection as the Temple. Some thought that a consecrated building, famous throughout the whole human race, ought not to be destroyed; that were it preserved it would be a memorial of Roman moderation; that its overthrow would leave an indelible mark of cruelty. But on the other hand, some, and Titus himself among them, were of opinion that the Temple, more than anything else, must be destroyed, that so the Jewish and the Christian superstitions might be thoroughly eradicated. These superstitions, though mutually opposed, had had their origin in the same people; the Christians had risen up from among the Jews; if the root was removed, the stem would soon perish." In reading the history of the Roman empire the student of sociology must always bear in mind one great and all-important fact, that the empire was created from the beginning, and afterwards sustained, by the power of the sword. No such empire, as we have before remarked, is ever like to curse again the world. Militarism is, perhaps, more prevalent at the present moment than it has ever been before in the history of the world. But there is no central all-embracing political power to wield its resources. Therefore it seems to follow that, if ever we have another cosmopolitanism such as that which for a time prevailed in the Roman world, it must be founded not upon force, but upon sympathy. A true sociology is only possible through a slow and steady growth, and can never be attained by any system of mechanical manipulation or political adjustment, however subtle and ingenious it may appear to be. There must be union before there can be unity. It is a remarkable and most interesting historical fact that the three nations of antiquity who, as we have said before, have most influenced modern thought and modern civilisation generally, seemed to lose their own individuality in proportion as they influenced the rest of the world. The Greeks, under Alexander the Great, permanently influenced the whole of the East, from Asia Minor to India; but in the process the original Greek States themselves sank into insignificance and degeneracy, and finally lost even the shadow of freedom. The same fate happened to Rome. The city became merged in the world—the "urbs" in the "orbs." Finally the Jewish State, the most narrow, exclusive, and the most firmly welded together of all, as within it race and religion were identical, lost its political existence altogether on the advent of Christianity; and although the race and the religion still remain, the political organisation seems to have departed for ever. It would seem as if nations, like individuals, must first efface themselves if they wish to exercise an abiding influence upon mankind. This great truth must be always borne in mind by the student of sociology. Modern civilisation, in spite of its standing armies, its international jealousies, its differences of race, religion, and language, is tending towards a new and a greater and more permanent cosmopolitanism than was ever reached by Rome. We shall again revert to this subject in the concluding article of this series.

